# Body Composition and Metabolic Profiles in Young Adults: A Cross-Sectional Comparison of People Who Use E-Cigarettes, People Who Smoke Cigarettes, and People Who Have Never Used Nicotine Products

**DOI:** 10.3390/jcm14134459

**Published:** 2025-06-23

**Authors:** Joanna Chwał, Hanna Zadoń, Piotr Szaflik, Radosław Dzik, Anna Filipowska, Rafał Doniec, Paweł Kostka, Robert Michnik

**Affiliations:** 1 Department of Medical Informatics and Artificial Intelligence, Faculty of Biomedical Engineering, Silesian University of Technology, 41-800 Zabrze, Poland; anna.filipowska@polsl.pl (A.F.); rafal.doniec@polsl.pl (R.D.); pawel.kostka@polsl.pl (P.K.); 2 Joint Doctoral School, Silesian University of Technology, 44-100 Gliwice, Poland; 3 Department of Clinical Engineering, Academy of Silesia, 40-555 Katowice, Poland; radoslaw.dzik@akademiaslaska.pl; 4 Department of Biomechatronics, Faculty of Biomedical Engineering, Silesian University of Technology, 41-800 Zabrze, Poland; hanna.zadon@polsl.pl (H.Z.); piotr.szaflik@polsl.pl (P.S.); robert.michnik@polsl.pl (R.M.)

**Keywords:** e-cigarettes, metabolic health, body composition, nicotine, bioelectrical impedance analysis, young adults, smoking, adiposity

## Abstract

**Background**: Recent research highlights uncertainties surrounding the metabolic effects of nicotine in young adults, particularly among people who use e-cigarettes. While traditional smoking is known to alter body composition, the metabolic impact of using e-cigarettes remains less understood. **Methods**: In this cross-sectional study, body composition (via bioelectrical impedance analysis) and lifestyle data were collected from 60 university students (mean age: 21.7 ± 1.9 years), who were classified as people who use e-cigarettes exclusively, people who smoke cigarettes exclusively, or people who have never used nicotine products. To address confounding by sex and age, inverse probability of treatment weighting (IPTW) was applied. **Results**: After adjustment, people who use e-cigarettes had significantly higher body fat percentage compared to people who have never used nicotine (β = 5.45, *p* = 0.001), while no significant differences were found between people who smoke cigarettes and other groups. Energy drink consumption was also positively associated with body fat percentage and metabolic age. Machine learning models, particularly k-nearest neighbors, achieved moderate classification accuracy (up to 72%) in distinguishing people who use nicotine from people who have never used nicotine based on physiological and lifestyle features. **Conclusions**: It is important to note that the majority of participants were metabolically healthy, and the observed differences occurred within a clinically normal range. While these findings suggest associations between e-cigarette use and higher adiposity in young adults, no causal inferences can be made due to the observational design. Further longitudinal studies are needed to explore the potential metabolic implications of nicotine use.

## 1. Introduction

Tobacco smoking stands as a major preventable cause of chronic diseases and premature deaths throughout the world [1]. Cigarette smoking produces significant metabolic effects in addition to its established cardiovascular and respiratory dangers. Research evidence shows that smoking leads to higher rates of metabolic syndrome and type 2 diabetes development [2]. The appetite-suppressant and thermogenic properties of nicotine result in lower body weight and BMI among people who smoke cigarettes when compared to people who have never used nicotine products [3]. The nicotine found in cigarettes activates neurotransmitters that decrease hunger while increasing resting energy expenditure, leading to weight reduction [1,4,5]. The fact that people who smoke cigarettes weigh less does not mean they have better metabolic health. People who smoke tend to develop more abdominal (visceral) fat even though they maintain a lower weight. The growth of central adiposity presents a major risk factor because it leads to insulin resistance, dyslipidemia, and elevated cardiometabolic risk. People who smoke cigarettes develop an unhealthy lean body composition profile, which includes reduced lean mass and abnormal fat distribution patterns that increase their risk for adverse metabolic outcomes even among young adults [6,7].

Electronic cigarettes (e-cigarettes) have become popular tobacco alternatives since their emergence two decades ago, especially among younger individuals [1,8]. The battery-powered devices produce inhalable vapor that contains nicotine without burning tobacco. E-cigarettes serve as a less harmful alternative to traditional cigarettes, thus attracting people who smoke and want to decrease their tobacco consumption [9]. Although e-cigarettes do not contain tobacco leaves or undergo combustion, they deliver nicotine in vaporized form. This distinction is important when comparing their physiological impact to that of traditional cigarettes. The metabolic consequences of using e-cigarettes on a long-term basis continue to be unknown. The majority of e-cigarettes deliver significant nicotine amounts, which could lead people who use them to experience weight-modulating effects similar to those of traditional cigarettes. Research indicates that people who use e-cigarettes share similar lower average BMI levels with people who smoke cigarettes [1,6]. The observed lower BMI in people who use e-cigarettes might stem from nicotine-induced appetite reduction and elevated metabolic rate, which resemble people who smoke cigarettes characteristics. The use of e-cigarettes has been linked to possible metabolic complications. Studies have found that using e-cigarettes leads to specific metabolic syndrome features, including central obesity and elevated blood pressure [2]. The ongoing research into e-cigarette health effects requires clarification about whether using e-cigarettes replaces cigarette use to reduce metabolic problems or simply changes their nature.

Body composition functions as a vital element of metabolic health, which shows different responses based on smoking habits. The distribution between fat and lean mass within normal body weight determines metabolic risk to a significant extent. The combination of body fat accumulation with decreased muscle mass, which some people who smoke cigarettes develop, leads to insulin resistance and dyslipidemia [6,7]. The assessment of body composition together with metabolic indicators in people who smoke cigarettes, people who use e-cigarettes, and people who have never used nicotine products enables researchers to detect the initial health impacts of these behaviors. Bioelectrical impedance analysis (BIA) serves as a useful noninvasive tool to measure body composition during population research. The harmless electrical current measurement through BIA enables fast calculations of fat mass, lean body mass, and total body water by determining the body’s electrical resistance [10]. BIA technology enables researchers to identify minimal variations in body fat distribution, muscle size, and basic metabolic rates between people with varying lifestyle patterns. The combination of BIA data with lifestyle questionnaire results enables researchers to evaluate the impact of smoking behaviors on body composition and metabolic state among healthy young adults.

Building upon our previous research, which employed machine learning techniques to identify demographic, familial, and social predictors of smoking and e-cigarette use among young adults [11], this study shifts focus toward physiological outcomes—specifically the metabolic profile associated with e-cigarette use.

The increasing popularity of e-cigarettes alongside ongoing smoking health issues requires researchers to analyze their effects on metabolic health indicators. Research about body composition variations between people who smoke traditional cigarettes, people who use e-cigarettes, and people who have never used nicotine products remains scarce, especially when studying university students. This research aimed to analyze body composition and metabolic indicators between conventional people who smoke cigarettes, people who use e-cigarettes, and people who have never used nicotine products among Silesian University of Technology students. The research combines BIA measurements with lifestyle questionnaires to explore whether using e-cigarettes is associated with differences in body fat distribution and metabolic profiles when compared to smoking, and to establish fundamental evidence about the metabolic effects of cigarette and e-cigarette use in young adults. The research results will provide essential knowledge about how tobacco and nicotine products affect early indicators of metabolic health.

## 2. Materials and Methods

The study protocol was designed to examine the association between nicotine/tobacco use status and selected anthropometric and metabolic parameters in a university student population. To achieve this objective, comprehensive data were collected through standardized body composition measurements and structured lifestyle questionnaires. The following subsections provide detailed descriptions of the participants, procedures, and statistical methods used in the analysis.

### 2.1. Participants and Data Collection

Young adult volunteers from the Faculty of Biomedical Engineering at the Silesian University of Technology in Gliwice, Poland, recruited through student mailing lists, on-campus advertisements, and classroom announcements, made up the study population. The research participants joined the study through convenience sampling during the academic year 2024/2025. The study included participants who were older than 18 years old and obtained necessary consent before joining the research. The Ethics Committee for Research Involving Human Participants at the Silesian University of Technology in Gliwice, Poland approved this study through resolution No. 3/2025 on 11 March 2025. The participants received group assignments according to their self-reported tobacco consumption habits between traditional cigarettes (T), electronic cigarettes (E), and none (N). The study did not use biochemical validation methods such as cotinine testing.

Anthropometric and body composition data were collected using a commercially available bioelectrical impedance analysis (BIA) device, the Tanita MC-780 (Tanita Corp., Meguro-ku, Japan). Measurements included body weight (kg), height (cm), body mass index (BMI), total body water (TBW), visceral fat level, muscle mass, fat mass, and metabolic age, among others. All measurements were taken under standardized conditions. Measurements were conducted barefoot and in light clothing. For each participant, the measurement protocol was conducted by a trained examiner following the manufacturer’s guidelines.

The participants filled out a validated lifestyle questionnaire to measure their dietary behavior, beverage consumption, physical activity, and sedentary time. In addition, they were asked to report their average number of daily smoking or e-cigarette use sessions. For the purpose of standardization, one e-cigarette session was defined as approximately 15 inhalations or 10 min of use. Only participants who reported regular use (more than 1 year) and more than 10 sessions per day were included in the analysis. For people who smoke cigarettes, one session was considered equivalent to smoking one full cigarette. Participants in this group also reported more than 10 cigarettes per day and had a smoking history of over one year. Based on self-reported nicotine use, respondents were classified into three groups. People who have never used nicotine products were defined as those who reported no current or past use of any nicotine or tobacco products, including even occasional use. People who smoke cigarettes were defined as those who exclusively smoked combustible cigarettes for at least one year, with no history of e-cigarette use. People who use e-cigarettes were defined analogously, as individuals who exclusively used e-cigarettes for at least one year without any use of traditional cigarettes. Those reporting dual use, or use of either product for less than one year, were excluded to reduce heterogeneity and isolate the effects of sustained, single-product use.

The questionnaire data were recorded through structured fields and manually checked for completeness. The final dataset was compiled in a spreadsheet format (Excel “.xlsx”, Microsoft Excel, Office 365, version 16.98 (25060824) for Mac), comprising both continuous and categorical variables. All identifying information was pseudonymized before analysis to ensure confidentiality.

### 2.2. Variable Selection

The dataset consisted of both numerical (continuous) and categorical variables. A total of 21 numerical variables were initially considered for analysis, including anthropometric measurements (e.g., body weight, height, BMI), body composition parameters (e.g., fat mass, visceral fat level, skeletal muscle mass, phase angle, total body water, extracellular water, intracellular water), metabolic indicators (e.g., metabolic age, basal metabolic rate), and selected lifestyle factors (e.g., daily sitting time, number of meals consumed per day, daily water intake, number of cups of coffee consumed per day, and frequency of energy drink consumption).

Eight categorical variables were included, covering demographic and lifestyle information such as sex, place of residence (urban vs. rural), occupational status, level of physical activity, declared dietary habits (e.g., regularity of meals, type of diet), transportation habits (e.g., commuting mode), and methods of food preparation (e.g., cooking vs. processed food).

Prior to analysis, numerical variables were converted to a double-precision floating-point format to enable statistical calculations. Categorical variables were encoded using embedded in Matlab software (Matlab 2024b, The Mathworks Inc., Natick, MA, USA) ‘categorical’ data type to facilitate proper handling in statistical tests and classification models. Missing data were assessed, and observations containing missing or non-convertible entries for critical variables were excluded listwise from multivariate analyses (e.g., principal component analysis, classification).

Variable selection for classification models was based on initial feature importance derived from a decision tree classifier, with the top-ranked variables subsequently used for model building and validation. In particular, eight features with the highest predictive value were selected for the final classification analysis.

### 2.3. Group Coding and Preprocessing

Participants were assigned to groups based on their self-assessment of smoking/using e-cigarette habits: people who smoke conventional cigarettes (T), people who use electronic cigarettes (E), and people who have never used nicotine products (N). The participant identity codes identified group membership using their suffixes (‘\_T’ for people who smoke conventional cigarettes, ‘\_E’ for people who use e-cigarettes, and no suffix for people who have never used nicotine products), which were validated by structured questionnaire replies. The classification model combined participants from groups T and E into a single “smoking” group labeled ‘1’, while people who have never used nicotine products (group N) received the label ‘0’. The re-coding allowed researchers to evaluate variables that distinguish people who use nicotine from people who have never used nicotine products using future classification models.

Prior to analysis, all numerical variables imported from the spreadsheet were converted to a double-precision floating-point format to enable proper statistical processing. Categorical variables were encoded using Matlab’s categorical data type to ensure compatibility with statistical tests and machine learning algorithms. Records containing missing or non-convertible entries for critical variables were excluded from analyses using a listwise deletion approach. Numerical variables were standardized where required, particularly in multivariate analyses such as principal component analysis. Finally, group labels were encoded as categorical variables to support consistent handling across classification tasks. All preprocessing steps were conducted to guarantee data integrity, minimize bias due to missing information, and optimize the performance of the statistical and machine learning models employed in the study.

### 2.4. Statistical Analysis

All statistical analyses were performed using Matlab R2024b. Prior to comparative testing, the distribution of continuous variables was assessed using the Lilliefors test for normality. The majority of variables showed significant deviations from normality (*p* < 0.05) so non-parametric statistical methods were used for analysis.

The Kruskal–Wallis H test served as a non-parametric replacement for one-way ANOVA to evaluate continuous variable differences between T, E, and N groups. The Mann–Whitney U test was used to conduct post-hoc comparisons between specific groups after detecting a statistically significant overall effect (*p* < 0.05). The *p*-values needed adjustment through Bonferroni correction because of multiple comparisons risk (*n* = 3) to prevent Type I errors. The Chi-square test of independence served to analyze differences between groups for categorical variables. The analysis used a two-tailed *p*-value threshold of less than 0.05 for statistical significance, while applying Bonferroni correction when necessary.

The analysis presented descriptive statistics through median values and interquartile ranges (IQRs) for continuous variables together with categorical variable data presented as counts and percentages. The analysis excluded cases with missing data by using listwise deletion to only include complete cases. The distribution of continuous variables across groups was displayed through boxplots, while contingency tables were used to present categorical variables.

The study presented descriptive statistics through median values and interquartile ranges (IQRs) for continuous data and counts with percentages for categorical data. The analysis excluded cases with missing data through listwise deletion, so only complete cases were used for comparative evaluations. The distribution of continuous variables across groups was displayed through boxplots and contingency tables were used to present categorical variables.

Due to differences in covariates across groups, particularly sex distribution, a propensity score weighted analysis was used to examine adjusted differences in body fat percentage. The analysis focused on three pairwise comparisons: (1) people who use e-cigarettes vs. people who have never used nicotine products, (2) people who use e-cigarettes vs. people who smoke conventional cigarettes, and (3) people who smoke cigarettes vs. people who have never used nicotine products. For each comparison, propensity scores were estimated using logistic regression based on sex and age. Inverse probability of treatment weighting (IPTW) was then applied to create a pseudo-population with balanced covariate distributions. Weighted linear regression models were used to estimate differences in body fat percentage between groups.

To evaluate the effectiveness of inverse probability of treatment weighting (IPTW), covariate balance was assessed using standardized mean differences (SMD) for sex and age. A threshold of SMD < 0.1 was considered acceptable. Separate diagnostics were conducted for each pairwise comparison (people who use e-cigarettes vs. people who have never used nicotine products, people who use e-cigarettes vs. people who smoke cigarettes, and people who smoke cigarettes vs. people who have never used nicotine products).

### 2.5. Correlation Analysis

The analysis of correlation between continuous variables used correlation analysis methods. The study calculated two types of correlation coefficients, which included the Pearson product-moment correlation coefficient for linear relationships between normally distributed variables and the Spearman rank correlation coefficient for non-parametric analysis of ordinal or non-normal variables. The Lilliefors test revealed most variables deviated from normality, so researchers focused their interpretation on Spearman correlations. The research produced correlation matrices for both Pearson and Spearman coefficients to evaluate statistical significance between each pair of variables. The study used a *p*-value threshold of 0.05 to establish statistical significance. The correlation structure was visualized through heatmaps that displayed correlation coefficients by their magnitude and direction through color coding.

In addition to the general correlation matrices, targeted correlation analyses were performed between selected lifestyle variables (e.g., sedentary time, coffee intake, energy drink consumption) and physiological parameters such as body mass index (BMI), basal metabolic rate (BMR), and metabolic age. The strength and direction of these associations were evaluated, and where appropriate, scatter plots were generated to illustrate significant findings. All correlation analyses were conducted using built-in functions from the Matlab Statistics and Machine Learning Toolbox.

### 2.6. Classification and Feature Importance

To evaluate the ability of physiological and lifestyle variables to discriminate between people who use nicotine and people who have never used nicotine products, several supervised classification models were developed. The rationale for including this approach was to complement group-based comparisons with data-driven pattern recognition across multiple variables, such as body composition metrics, physical activity, and dietary habits. Unlike traditional hypothesis testing, ML enables the detection of complex, potentially non-linear associations between input features and classification outcomes.

An initial attempt was made to classify participants into three groups: people who have never used nicotine products, people who smoke cigarettes exclusively, and people who use e-cigarettes exclusively. However, model performance was suboptimal, with high misclassification rates between the two groups of people who use nicotine. This outcome was attributed to overlapping physiological characteristics and unequal sample sizes. To address this limitation, a binary classification approach was adopted, where participants using either traditional cigarettes or electronic cigarettes were combined into a single people who use nicotine group (label = 1), and people who have never used nicotine products were labeled as 0. Initially, a decision tree classifier (CART algorithm) was trained using the entire set of continuous variables. Feature importance scores were extracted based on the reduction of the Gini impurity criterion at each split node. The eight most important features were subsequently selected for further model development to reduce dimensionality and minimize the risk of overfitting. Classification models were trained using the selected top eight variables and included a Decision Tree classifier, a Random Forest classifier consisting of 100 trees, and a k-Nearest Neighbors (kNN) classifier with k = 5.

Model performance was evaluated using 5-fold cross-validation to assess generalization ability. Accuracy was calculated as the proportion of correctly classified instances. Confusion matrices were generated to provide detailed insights into classification performance, including sensitivity and specificity. All classification analyses were performed using Matlab’s Statistics and Machine Learning Toolbox. Hyperparameters were selected based on standard defaults without extensive tuning, reflecting an exploratory approach rather than model optimization.

## 3. Results

The sixty participants in the final analysis were split into three groups: twenty were people who smoke cigarettes (Group T), twenty were people who use e-cigarettes (Group E), and twenty were people who have never used nicotine products (Group N). All participants had an average body mass index (BMI) of 22.3 kg/m^2^, with a median age of 22 years. Height, weight, body fat percentage, visceral fat level, skeletal muscle mass, total body water, and metabolic age are among the participants’ physiological and demographic details shown in Table 1. There were no differences between the groups in terms of home location or sex distribution. For continuous variables, the study used medians and interquartile ranges (IQRs); for categorical variables, it used counts and percentages to provide descriptive statistics.

**Figure 1:**
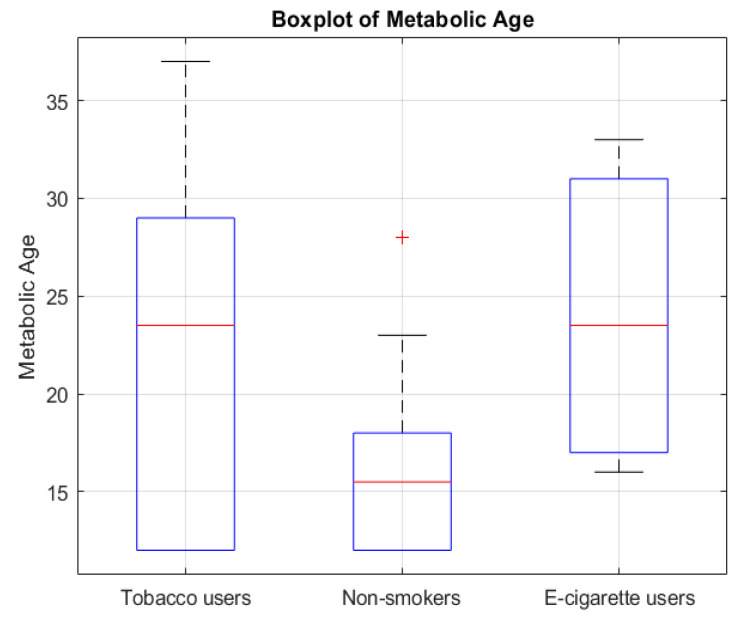
Boxplot of metabolic age across smoking/using e-cigarettes status groups. The red line represents the median. The red “+” symbol indicates an outlier.

**Figure 2:**
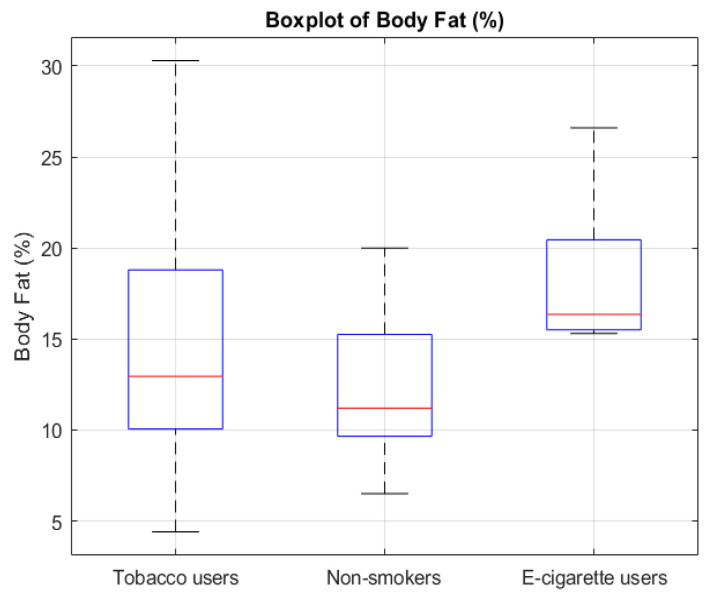
Boxplot of body fat percentage across smoking/using e-cigarettes status groups. The red line represents the median.

**Figure 3:**
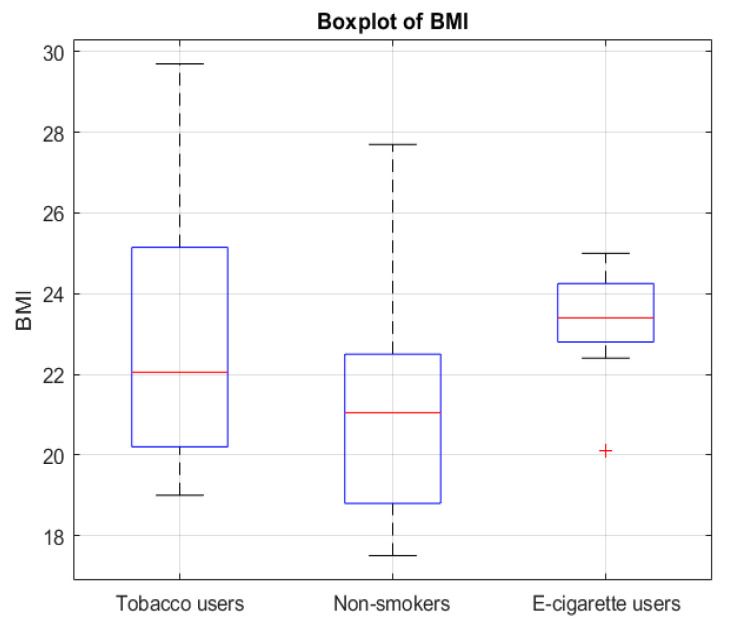
Boxplot of BMI across smoking/using e-cigarettes status groups. The red line represents the median. The red “+” symbol indicates an outlier.

**Figure 4:**
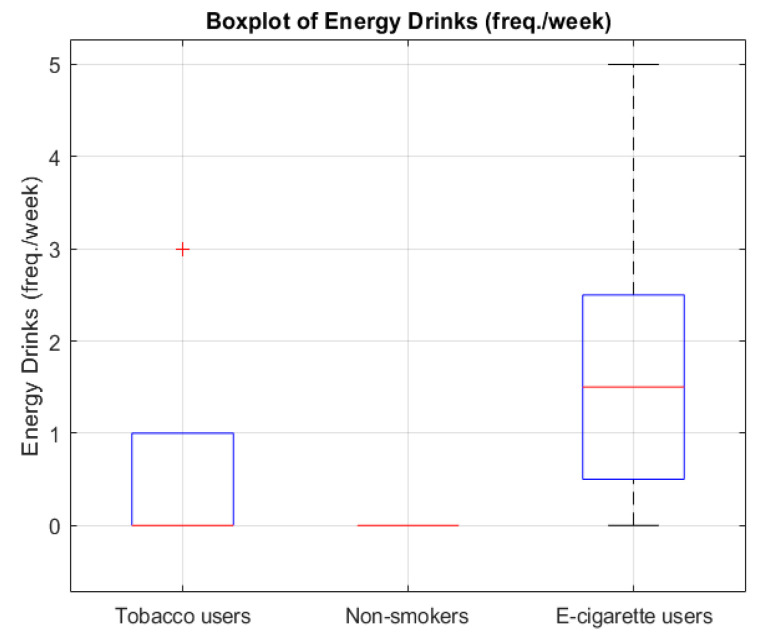
Boxplot of energy drink consumption frequency across smoking/using e-cigarettes status groups. The red line represents the median. The red “+” symbol indicates an outlier.

The analysis showed that groups differed significantly regarding metabolic age (*p* = 0.0429) (Figure 1), body fat percentage (*p* = 0.0203) (Figure 2), BMI (*p* = 0.0295) (Figure 3), and the number of energy drink consumed (*p* = 0.0007) (Figure 4). The results of the Mann–Whitney U test with Bonferroni correction showed that people who use e-cigarettes had higher BMI (*p* = 0.0199), higher body fat percentage (*p* = 0.0127), and higher metabolic age (*p* = 0.0248) than people who have never used nicotine products.

According to the data, energy drink consumption was higher among people who use e-cigarettes than among people who smoke cigarettes and people who have never used nicotine products (*p* = 0.0480 and *p* = 0.0011, respectively). There were no differences in these characteristics between the people who smoke cigarettes and the other groups after controlling for multiple comparisons.

After controlling for multiple comparisons, there were no discernible differences in these variables between the people who smoke cigarettes and the other groups. Table 2 offers a thorough summary of the comparisons, complete with *p*-values from post-hoc analyses and Kruskal–Wallis tests.

The boxplot presents the distribution of metabolic age in people who smoke cigarettes, people who use e-cigarettes, and people who have never used nicotine products. The median, interquartile range (IQR), and outliers are shown. A significant difference was observed between people who use e-cigarettes and people who have never used nicotine products (*p* = 0.0248, Bonferroni corrected).

The boxplot illustrates the differences in body fat percentage among people who smoke cigarettes, people who use e-cigarettes, and people who have never used nicotine products. People who use e-cigarettes exhibited significantly higher body fat percentage compared to people who have never used nicotine products (*p* = 0.0127, Bonferroni corrected).

The boxplot shows the distribution of BMI values among people who smoke cigarettes, people who use e-cigarettes, and people who have never used nicotine products. A significant increase in BMI was found in people who use e-cigarettes compared to people who have never used nicotine products (*p* = 0.0199, Bonferroni corrected).

The figure presents the frequency of energy drink consumption per week among the three groups. People who use e-cigarettes reported significantly higher consumption compared to both people who have never used nicotine products (*p* = 0.0011) and people who smoke cigarettes (*p* = 0.0480).

**Figure 5:**
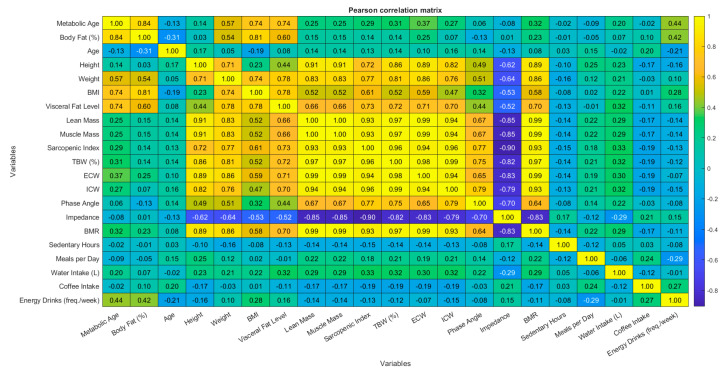
Pearson’s correlation matrix between continuous variables, (*p* < 0.05).

**Figure 6:**
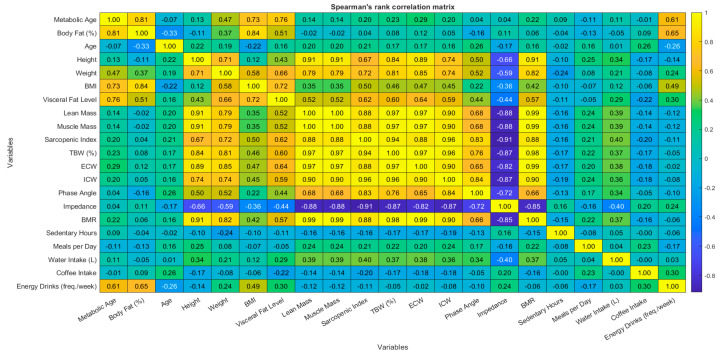
Spearman’s rank correlation matrix between continuous variables, (*p* < 0.05).

Correlation analyses were conducted using both Pearson’s and Spearman’s methods to examine the relationships between continuous variables. The majority of variables demonstrated consistent patterns across both methods. The Pearson correlation analysis revealed that body weight demonstrated strong positive relationships with muscle mass (r = 0.83), total body water (r = 0.81), and body fat percentage (r = 0.54). BMI showed positive relationships with body fat percentage (r = 0.81) and visceral fat level (r = 0.78). Body fat percentage and visceral fat levels showed strong correlations with metabolic age (r = 0.84 and r = 0.74, respectively). The number of energy drinks consumed per week demonstrated moderate positive associations with metabolic age (r = 0.61, *p* < 0.001) and body fat percentage (r = 0.65, *p* < 0.001). The results from Spearman’s rank correlation coefficients validated the observed patterns. Body weight maintained a strong positive relationship with muscle mass, total body water, and body fat percentage measurements. The results from Spearman’s rank correlation coefficients confirmed the previously identified relationships between energy drink consumption frequency and metabolic age (ρ = 0.61) and body fat percentage (ρ = 0.65). The heatmaps in Figure 5 and Figure 6 present a visual representation of the correlation strengths and directions between variables. The diagrams use *p* < 0.05 as the threshold to mark significant associations. The heatmaps display yellow for positive correlations and blue for negative correlations, where darker shades represent stronger negative relationships and lighter shades represent stronger positive relationships.

The research included both correlation matrices and specific pairwise correlation tests to study the connections between certain lifestyle elements and body composition and metabolic results. The analysis revealed no meaningful relationships between daily sedentary time and BMI (r = −0.10, *p* = 0.5615), basal metabolic rate (BMR) (r = −0.15, *p* = 0.3819), metabolic age (r = 0.09, *p* = 0.6025), or body fat percentage (r = −0.04, *p* = 0.8170). The analysis showed that coffee consumption did not correlate with BMI (r = −0.06, *p* = 0.7151), BMR (r = −0.16, *p* = 0.3499), metabolic age (r = −0.01, *p* = 0.9357), or body fat percentage (r = 0.09, *p* = 0.5850). The analysis revealed that energy drink consumption had statistically significant positive relationships with BMI (r = 0.49, *p* = 0.0025), metabolic age (r = 0.61, *p* = 0.0001), and body fat percentage (r = 0.65, *p* < 0.0001), which indicates that regular energy drink consumption might be associated with negative metabolic effects.

To address baseline differences in covariates, particularly sex and age, a series of propensity score weighted (PSW) linear regression models were used to estimate adjusted differences in body fat percentage between groups. In the comparison between people who use e-cigarettes and people who have never used nicotine products, the model showed a statistically significant difference in body fat percentage, with people who use e-cigarettes having higher levels than people who have never used nicotine products (β = 5.45, *p* = 0.001, R^2^ = 0.38). This result remained consistent after weighting for sex and age using inverse probability of treatment weighting (IPTW). In contrast, no statistically significant differences were observed between people who use e-cigarettes and people who smoke cigarettes (β = 1.91, *p* = 0.39, R^2^ = 0.03), nor between people who smoke cigarettes and people who have never used nicotine products (β = 2.06, *p* = 0.39, R^2^ = 0.03). These results (Table 3) suggest that the elevated body fat observed in people who use e-cigarettes is not meaningfully different from that of people who smoke cigarettes when controlling for sex and age and may reflect general associations with nicotine exposure.

**Figure 7:**
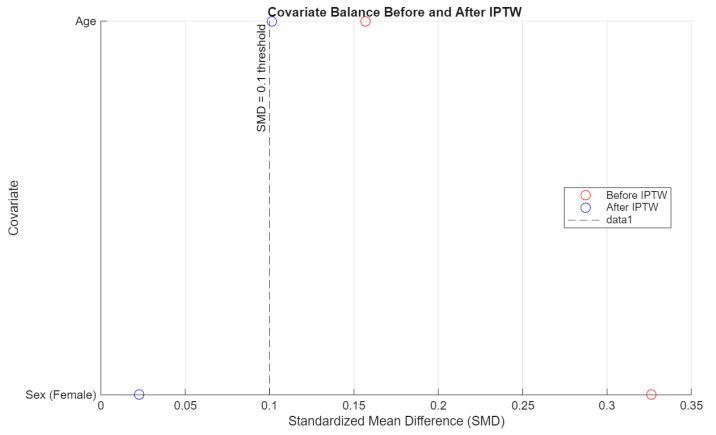
Covariate balance: people who smoke cigarettes vs. people who have never used nicotine products.

**Figure 8:**
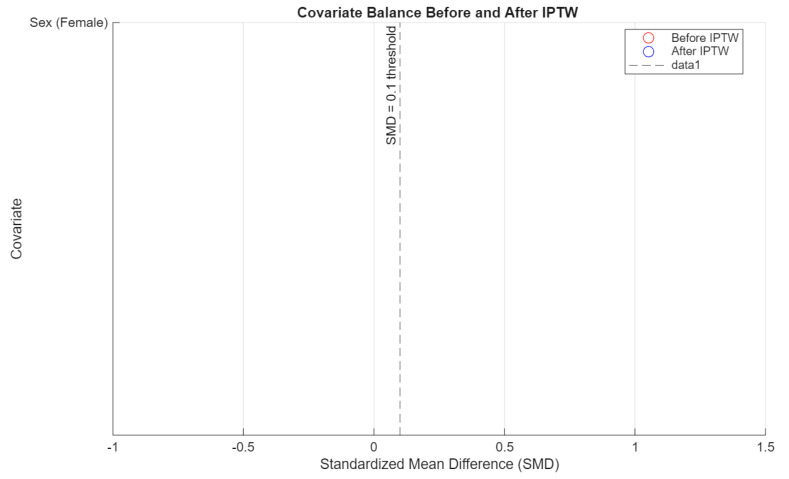
Covariate balance: people who use e-cigarettes vs. people who have never used nicotine products.

**Figure 9:**
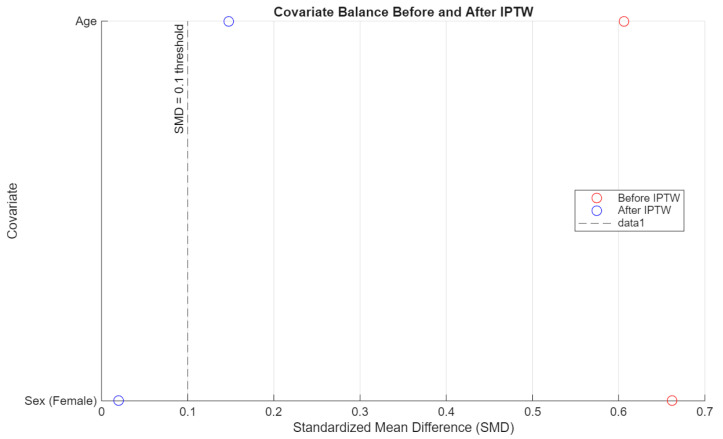
Covariate balance: people who use e-cigarettes vs. people who smoke cigarettes.

The IPTW procedure improved covariate balance across all comparisons. In particular, the standardized mean difference (SMD) for sex was reduced to near-zero levels in all pairwise contrasts, while age balance improved and met the conventional threshold (SMD < 0.1) in both comparisons between people who use e-cigarettes and people who have never used nicotine products, and between people who use e-cigarettes and people who smoke cigarettes. These results support the adequacy of the weighting procedure (Figure 7, Figure 8 and Figure 9), though some degree of residual confounding cannot be entirely excluded. There is also the possibility of residual confounding, as the propensity score weighting model included only age and sex. Notably, the small number of male participants in the e-cigarette group may limit the ability to fully control for sex differences in the analysis, particularly given the strong confounding effect of sex observed in unadjusted comparisons. Other potentially relevant confounders such as socioeconomic status, dietary habits, physical activity, or psychological stress were not available for inclusion and may have influenced the results.

**Figure 10:**
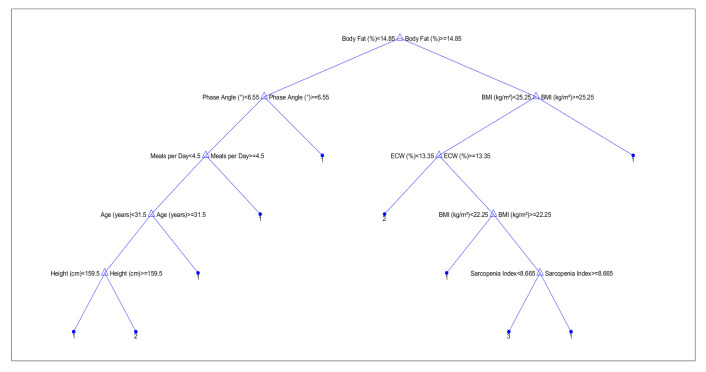
Decision Tree structure for classification of smoking/using e-cigarettes status. The numbers at the terminal nodes indicate the predicted class labels.

**Figure 11:**
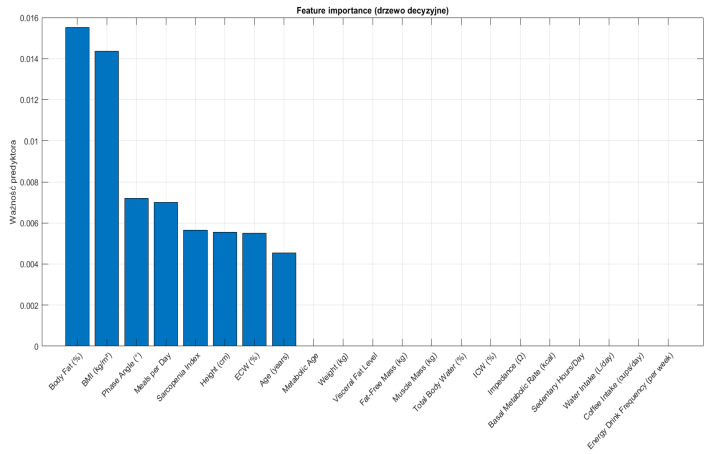
Feature importance ranking from the Decision Tree model.

Supervised machine learning models were developed to classify participants based on their smoking/using e-cigarette status, in order to distinguish between people who use nicotine (those who smoke cigarettes and those who use e-cigarettes) and people who have never used nicotine products. Initially, a decision tree classifier (Figure 10) was built using all available continuous variables. The model was evaluated with 5-fold cross-validation to prevent overfitting. The initial decision tree yielded an accuracy of 50.0%, indicating a modest predictive capability. Feature importance analysis (Figure 11) revealed that metabolic age, body fat percentage, and BMI were the most influential variables.

**Figure 12:**
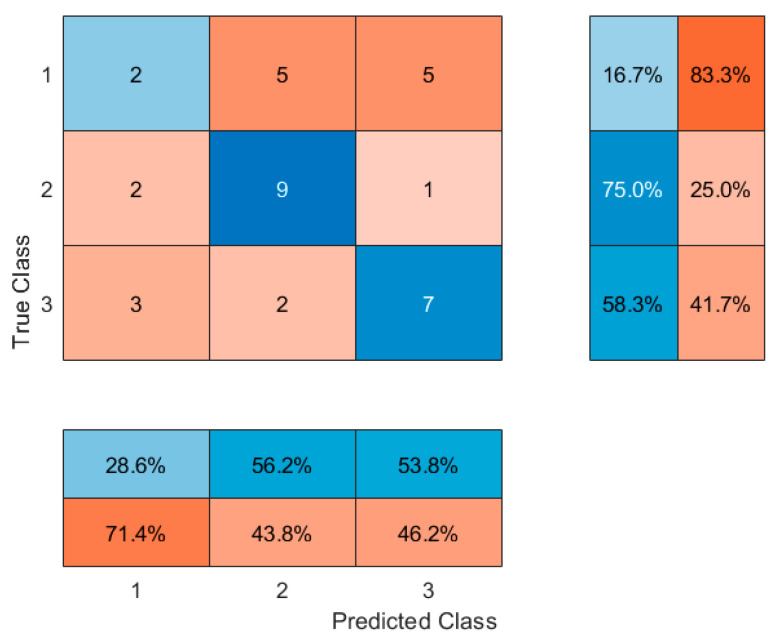
Confusion matrix for Decision Tree classifier for all features and 3 classes. 1—people who have never used nicotine products, 2—people who use e-cigarettes, 3—traditional cigarette people who smoke cigarettes. The central matrix shows the number of correctly and incorrectly classified instances (absolute counts). The right panel displays class-wise recall (sensitivity), and the bottom panel shows class-wise precision. Cell background colors represent the magnitude of values, from low (light blue) to high (dark red).

**Figure 13:**
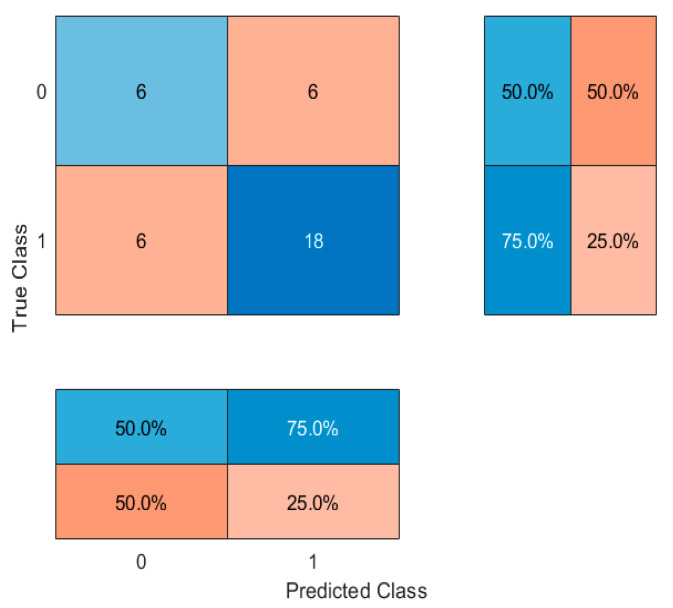
Confusion matrix for Decision Tree classifier for 8 features. 0—people who have never used nicotine products, 1—people who use nicotine altogether. The central matrix shows the number of correctly and incorrectly classified instances (absolute counts). The right panel displays class-wise recall (sensitivity), and the bottom panel shows class-wise precision. Cell background colors represent the magnitude of values, from low (light blue) to high (dark red).

**Figure 14:**
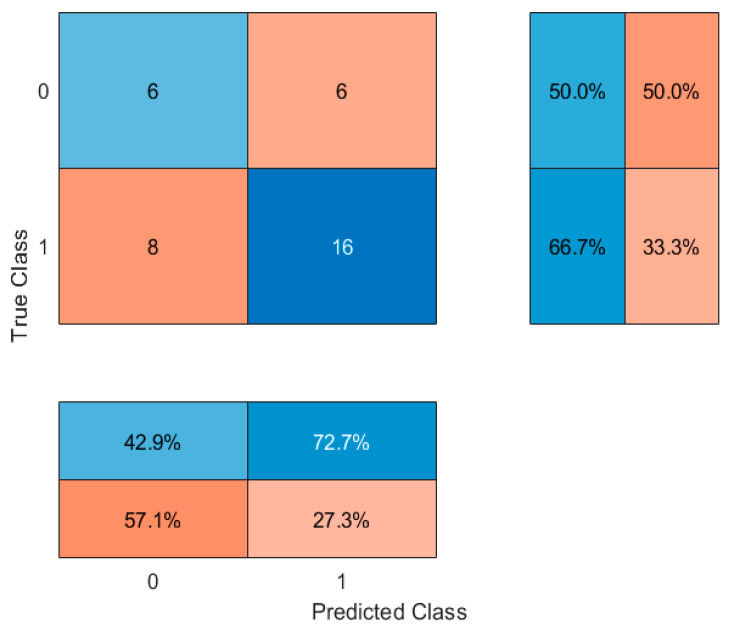
Confusion matrix for Random Forest classifier. 0—people who have never used nicotine products, 1—people who use nicotine altogether. The central matrix shows the number of correctly and incorrectly classified instances (absolute counts). The right panel displays class-wise recall (sensitivity), and the bottom panel shows class-wise precision. Cell background colors represent the magnitude of values, from low (light blue) to high (dark red).

**Figure 15:**
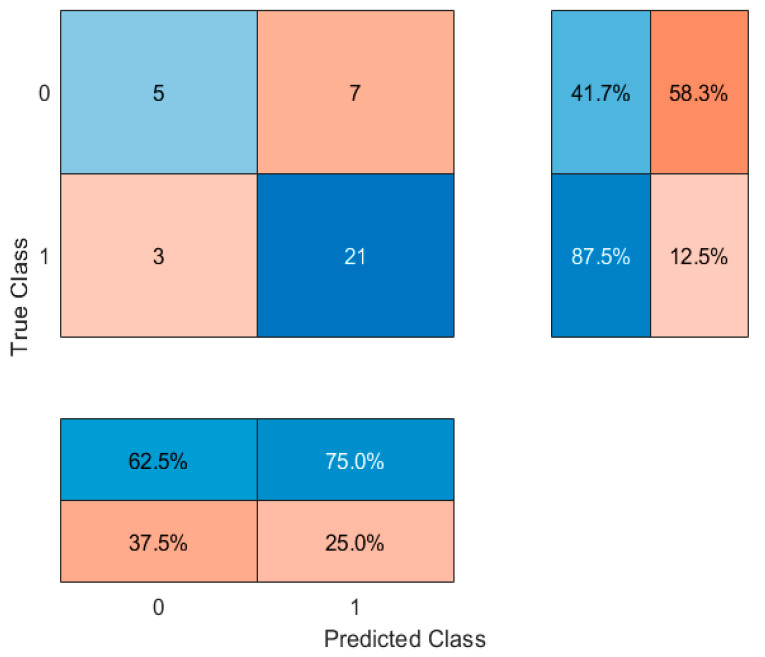
Confusion matrix for k-Nearest Neighbors (k = 5) classifier. 0—people who have never used nicotine products, 1—people who use nicotine altogether. The central matrix shows the number of correctly and incorrectly classified instances (absolute counts). The right panel displays class-wise recall (sensitivity), and the bottom panel shows class-wise precision. Cell background colors represent the magnitude of values, from low (light blue) to high (dark red).

Subsequently, models were refined by including only the top eight most important features. This approach slightly improved classification accuracy to 66.67% with the decision tree. Alternative classifiers were then explored. Random Forest (with 100 trees) achieved an accuracy of 61.1%, suggesting that an ensemble approach did not markedly improve performance. In contrast, a k-nearest neighbors (kNN) classifier (k = 5) provided a notable improvement, achieving an accuracy of 72.22%. These results (Table 4) suggest that a kNN classifier, based on selected anthropometric and lifestyle features, may provide a better tool for distinguishing between people who use nicotine and people who have never used nicotine products in young adult populations. Confusion matrices were generated and are shown in Figure 12, Figure 13, Figure 14 and Figure 15.

## 4. Discussion

The current research revealed that people who use e-cigarettes maintained greater body fat percentage, BMI, and metabolic age than people who have never used nicotine products, while people who smoke cigarettes showed no differences with people who have never used nicotine products in these measurements. The findings indicate that e-cigarette use was associated with less favorable body composition indicators in young adults. Although nicotine has been widely regarded as an appetite suppressant and is sometimes used for weight control, the present findings suggest that habitual e-cigarette use in young adults was associated with increased body fat percentage. However, it is important to note that the relationship between nicotine and metabolic outcomes is likely multifactorial and may be modulated by behavioral patterns, sex differences, and lifestyle characteristics. Therefore, our results do not negate the appetite-suppressing potential of nicotine in some contexts but rather emphasize that long-term use does not necessarily lead to lower adiposity. The observation that both people who smoke cigarettes and people who use e-cigarettes exhibited similar levels of metabolic disturbance suggests that nicotine exposure—regardless of delivery method—may be associated with comparable differences in metabolic indicators, assuming similar usage intensity. While nicotine intake was habitual in both groups, the patterns of consumption differed between people who smoke cigarettes and people who use e-cigarettes. Traditional cigarette smoking typically occurs in defined units (e.g., one cigarette), whereas e-cigarette use is often more fragmented, involving multiple short inhalation sessions throughout the day. These behavioral differences make the direct comparison of “amount of tobacco” unfeasible. Therefore, this study focused on frequency and regularity of use as the most practical and behaviorally relevant measure of nicotine exposure across both groups. It should be noted that all three groups demonstrated average BMI values within the clinically healthy range (22–23 kg/m^2^), and metabolic age estimates were close to participants’ chronological age. Thus, although statistically significant differences were observed, the overall metabolic health of the sample was within normative values.

While our inclusion criteria ensured that participants had used only one type of nicotine product for at least one year, we acknowledge that relying on self-reported data may not fully capture occasional or unreported prior use. Therefore, although people who have never used nicotine products were defined as having never used any nicotine products, and people who use nicotine were categorized based on exclusive and sustained use, the possibility of recall bias or misclassification cannot be entirely excluded. Given the cross-sectional design and self-selected exposure groups, the observed associations do not establish temporal or causal relationships. It remains unclear whether these metabolic differences preceded or followed the initiation of nicotine use.

Multiple recent studies involving comparable age groups validate our research findings. A cross-sectional study of Slovak young adults (mean age ~22 years) demonstrated that people who smoke cigarettes regularly possessed larger waist circumference measurements and higher BMI and fat mass values, including percent body fat and visceral fat area, compared to people who have never used nicotine products [8]. The research shows that people who smoke cigarettes in early adulthood tend to have greater total body fat and central fat distribution. The study by Radmilović et al. demonstrated that middle-aged people who smoke cigarettes displayed higher body fat percentages and their biological age exceeded their actual age, while people who have never used nicotine products maintained equivalent values [12]. The observed increase in “metabolic age” among people who use nicotine matches our findings and suggests that tobacco use over time speeds up metabolic aging. The research supports that people who smoke cigarettes typically experience poor weight and fat distribution patterns even though their weight changes are not always obvious.

The current research supports previous studies that establish links between using e-cigarettes and metabolic risks even though data about e-cigarette use and body composition remains limited. A Korean population-based study discovered that people who used e-cigarettes who were active at the time of the study had larger waist measurements and the highest risk of developing abdominal obesity and hypertriglyceridemia when compared to people who have never used nicotine products. The research showed that people who use e-cigarettes developed metabolic syndrome at higher rates than both people who smoke cigarettes exclusively and people who have never used nicotine products, with the people who use e-cigarettes currently showing the highest risk [13]. The epidemiological evidence supports our findings of increased metabolic age in people who use e-cigarettes because using e-cigarettes does not provide any health benefits and may actually cause harm to metabolic health and body composition. Research indicates that youth who use conventional cigarettes have the same BMI increases as those who use electronic cigarettes. Longitudinal research showed that overweight adolescents used tobacco products more often and electronic tobacco products and conventional tobacco products showed equal positive effects on BMI when used separately [14]. The traditional understanding of people who smoke cigarettes being lean based on older adult data does not apply because weight and nicotine use relationships differ across various population ages and factors.

The relationship between nicotine use and weight outcomes shows complexity because of behavioral elements at play. Young people who use e-cigarettes may experience higher adiposity because they tend to engage in multiple health-risk behaviors simultaneously. The e-cigarette group consumed significantly more energy drinks according to our research and similar patterns have been observed in other studies. The research by Falbová et al. (2018) showed that young adults who smoked had higher consumption of energy drinks, alcohol, and coffee while engaging in less physical activity [8]. The combination of behaviors that include consuming high-calorie sugary drinks and being sedentary may result in weight gain and increased body fat, which opposes the immediate appetite-suppressing effects of nicotine. Young people commonly believe that nicotine serves as a weight management tool. Research shows that overweight individuals tend to use e-cigarettes due to the belief that these devices will help them maintain their weight [13]. Young people who use e-cigarettes and people who smoke cigarettes demonstrate paradoxically higher BMI and body fat, according to multiple studies—including ours—despite their weight-control intentions. The short-term appetite suppression from nicotine appears to be outmatched by metabolic changes and compensatory behaviors such as increased snacking or sugary drink consumption. The observed weight gain following smoking cessation in clinical settings also occurs when people use e-cigarettes as cessation aids because nicotine replacement through using e-cigarettes does not fully duplicate all metabolic effects of smoking [15].

Chronic nicotine exposure creates conditions that lead to undesirable fat distribution patterns and metabolic problems even though it suppresses appetite. The combination of insulin resistance and elevated circulating cortisol levels caused by nicotine leads to increased visceral fat accumulation and dyslipidemia [16]. Research shows that young people who smoke cigarettes possess more visceral adipose tissue than people who have never used nicotine products who have the same BMI [8], and males who use e-cigarettes in this study had elevated triglyceride levels compared to people who have never used nicotine products [13]. The observed changes lead to an increased “metabolic age”, which indicates that the body functions older than its actual age when measuring basal metabolic rate and fat storage. The elevated metabolic age in people who use e-cigarettes indicates early signs of metabolic changes, including metabolic slowdown and reduced lean body mass compared to fat mass. These changes will eventually raise the probability of developing metabolic syndrome and cardiovascular disease. The study needs to consider the social environment through which youth consume e-cigarettes. The widespread use of e-cigarettes among university students stems from their belief that these devices are safer than traditional cigarettes, as well as their social approval and digital marketing strategies [17,18,19]. The mentioned factors tend to support patterns of use that combine with additional risky behaviors, including excessive energy drink consumption and inactive lifestyles [20,21,22,23]. Future research needs to use objective biomarkers such as cortisol and lipid profiles, inflammatory markers, and insulin resistance indices to fully understand metabolic dysregulation in people who use nicotine. Wearable devices that track physical activity and sleep patterns should be integrated to improve the precision of lifestyle data analysis for body composition results.

Our research expands existing studies that demonstrate that both smoking and using e-cigarettes create negative impacts on body composition and metabolic indicators among young individuals. The weight-suppressive effects of smoking observed in older adults do not apply to college-aged and adolescent populations according to this study and other research in these age groups. The study shows that people who use nicotine among young people tend to have increased body fat while maintaining their BMI levels and developing preliminary signs of metabolic problems. The e-cigarette group demonstrated higher adiposity and metabolic age, which suggests that using e-cigarettes may not be metabolically neutral, although causality cannot be inferred from the data. The risks using e-cigarettes pose to obesity-related conditions match or surpass those associated with traditional tobacco use. These findings demonstrate the necessity for additional research into how new nicotine delivery systems affect students’ physical health and support public health warnings that both smoking and using e-cigarettes are ineffective for weight control and body composition maintenance. The study provides vital information to campus health programs by showing that using e-cigarette intervention programs must address dietary and lifestyle factors that lead to excessive body fat among students. A detailed understanding of weight and metabolic health effects between people who use nicotine and people who have never used nicotine products among young adults is essential for creating effective prevention measures and disproving the misconception that using e-cigarettes has no negative impact on weight or metabolic health.

The research contains several limitations that need to be recognized. The cross-sectional study design makes it impossible to determine how smoking behaviors affect metabolic results. The study found associations between using e-cigarettes and increased body fat and metabolic age, but it does not show whether using e-cigarettes caused these changes or if people with poor body composition were more likely to use them. Additional longitudinal research is needed to determine the exact cause-and-effect relationship between these observed effects. The study’s findings have limited general applicability because it included a small group of biomedical engineering students from one technical university. The study results might not represent the entire population because they do not capture diverse socioeconomic groups and do not show how gender influences nicotine and lifestyle responses. Although biochemical markers of nicotine exposure were not collected, we attempted to control for exposure consistency by excluding people who use nicotine occasionally and quantifying daily session frequency through a standardized questionnaire. BIA provides a non-invasive body composition assessment, but its accuracy falls short of DEXA or MRI imaging techniques, especially when measuring visceral adipose tissue. BIA-derived metabolic age serves as a useful measure of physiological condition yet it represents a calculated value that does not measure actual metabolic function. The self-reported data from questionnaires about lifestyle habits contains potential biases from recall errors and social desirability biases. The accuracy of specific variables might be affected by these limitations especially when people fail to report their unhealthy behaviors. Another important limitation is the relatively small sample size (*N* = 60), which may limit the generalizability and statistical power of the findings. The modest number of participants may reduce the ability to detect subtle effects or interactions and limit the extent to which results can be extrapolated to broader populations. The machine learning models used for classification faced restrictions because of the dataset’s limited size and structure. The identified predictive patterns were limited by both the number of input variables and the class distribution, which resulted in modest model performance. Future research needs to analyze bigger datasets with diverse populations to enhance both predictive accuracy and model robustness.

## 5. Conclusions

This study provides new evidence regarding the differences in body composition and metabolic profiles among young adults who use e-cigarettes compared to those who do not use nicotine products. Participants who used e-cigarettes demonstrated higher body fat percentage, BMI, and metabolic age relative to individuals who reported no history of nicotine use. These differences were not observed among people who smoke cigarettes compared to people who have never used nicotine products. Additionally, people who use e-cigarettes reported higher energy drink consumption, which was positively associated with indicators of increased adiposity and metabolic age. These findings highlight potential patterns of co-occurring lifestyle behaviors that may be important in understanding overall metabolic risk.

The best-performing machine learning model that used k-nearest neighbors achieved 72.2% accuracy in differentiating between people who use nicotine and people who have never used nicotine products using physiological and lifestyle characteristics, although the models demonstrated moderate accuracy. The metabolic differences detected between people who use nicotine and people who have never used nicotine products were not fully explained by the model, which raises the possibility of behavioral hormonal or genetic factors as contributors to these differences.

Our findings highlight the need to further explore the potential relationships between e-cigarette use and indicators of metabolic health in young adults. The results suggest that e-cigarette use may be associated with differences in body composition and metabolic profiles that resemble or exceed those observed in people who smoke cigarettes. However, given the cross-sectional and observational nature of the study, no conclusions can be drawn regarding the direction or causality of these associations. Public health discussions around nicotine use should consider the broader set of lifestyle factors that tend to co-occur with e-cigarette use, rather than framing these products as metabolically safer alternatives. These findings contribute to the growing body of literature examining the health correlates of nicotine use in young populations and underscore the importance of further research and preventive efforts targeting overall health behaviors.

Given the study’s cross-sectional design, it is not possible to determine the temporal order of nicotine use and metabolic differences, and the observed associations may be influenced by unmeasured or insufficiently adjusted confounding factors, particularly the strong imbalance in sex distribution between groups, as well as other variables such as socioeconomic status, dietary patterns, or stress. Therefore, we do not interpret these findings as evidence for the causal effects of e-cigarette use on metabolic health. Instead, they should be viewed as a basis for generating hypotheses to be tested in future longitudinal and experimental studies that can better clarify the directionality and mechanisms underlying these associations.

## Figures and Tables

**Table 1:** Baseline characteristics of participants by group. Data are presented as median [interquartile range] for continuous variables and as number (percentage) for categorical variables.

Variable	All Participants (*n* = 60)	People Who Smoke Cigarettes (*n* = 20)	People Who Use E-Cigarettes (*n* = 20)	People Who Have Never Used Nicotine Products (*n* = 20)
Age (years)	22.0 [2.25]	22.0 [2.25]	20.5 [3.0]	22.0 [1.75]
Height (cm)	173.5 [16.25]	176.5 [8.0]	170.0 [12.25]	171.5 [20.75]
Weight (kg)	65.35 [13.35]	68.75 [20.15]	65.35 [4.8]	62.1 [21.08]
BMI (kg/m^2^)	22.3 [3.55]	22.05 [4.62]	23.4 [1.33]	21.05 [3.2]
Body fat (%)	15.35 [5.87]	12.95 [7.17]	16.35 [4.32]	11.2 [5.15]
Visceral fat level	2.0 [2.0]	2.0 [2.25]	2.0 [2.0]	1.0 [1.0]
Muscle mass (kg)	47.35 [17.7]	52.25 [15.88]	46.6 [10.2]	45.75 [20.67]
Total body water (TBW) (%)	32.1 [13.12]	39.7 [14.9]	31.05 [6.1]	30.85 [14.55]
Metabolic age (years)	18.0 [13.25]	23.5 [17.0]	23.5 [13.0]	15.5 [5.5]
Sex—Male (%)	15 (41.7%)	7 (58.3%)	2 (16.7%)	5 (41.7%)
Sex—Female (%)	21 (58.3%)	5 (41.7%)	10 (83.3%)	7 (58.3%)

**Table 2:** Group comparisons for continuous variables between smoking/using e-cigarettes status groups. Data are presented as *p*-values from Kruskal–Wallis tests. Significant pairwise comparisons were assessed using Mann–Whitney U tests with Bonferroni correction.

Variable	Kruskal–Wallis *p*-Value	Significant Comparisons (Bonferroni-Corrected *p*)	
Metabolic Age	0.0429	People who use e-cigarettes vs people who have never used nicotine products (*p* = 0.0248)	significant
Body Fat	0.0203	People who use e-cigarettes vs people who have never used nicotine products (*p* = 0.0127)	significant
BMI	0.0295	People who use e-cigarettes vs people who have never used nicotine products (*p* = 0.0199)	significant
Chronological Age	0.0721	People who use e-cigarettes vs people who have never used nicotine products (trend, *p* = 0.0938)	non-significant, trend
Energy Drink Consumption	0.0007	People who use e-cigarettes vs people who have never used nicotine products (*p* = 0.0011)	significant
		People who use e-cigarettes vs people who smoke cigarettes (*p* = 0.0480)	significant

**Table 3:** Results of propensity score weighted linear regression models comparing body fat percentage between groups.

Comparison	β (Body Fat %)	95% CI	*p*-Value	R^2^	n
People who use e-cigarettes vs. people who have never used nicotine products	5.45	[2.38, 8.52]	0.001	0.38	24
People who use e-cigarettes vs. people who smoke cigarettes	1.91	[−2.6, 6.4]	0.391	0.03	24
People who smoke cigarettes vs. people who have never used nicotine products	2.06	[−2.8, 6.9]	0.390	0.03	24

**Table 4:** Performance of machine learning classifiers in distinguishing people who use nicotine from people who have never used nicotine products.

Classifier	Features Used	Cross-Validated Accuracy (%)
Decision Tree	All features	50.0%
Decision Tree	Top 8 features	66.67%
Random Forest (100 trees)	Top 8 features	61.1%
k-Nearest Neighbors (k = 5)	Top 8 features	72.22%

## Data Availability

The authors confirm that the data supporting the findings of this study are available upon request. The dataset is not publicly accessible but can be shared with interested researchers upon reasonable request.
